# Solitary thin-walled cystic lung cancer with extensive extrapulmonary metastasis: A case report and review of the literature

**DOI:** 10.1097/MD.0000000000012950

**Published:** 2018-10-26

**Authors:** Xiang Wang, Yun-Xia Tao, Miao Zhang, Wen-Bin Wu, Dun-Peng Yang, Min Wang

**Affiliations:** a Thoracic Oncology Center; b Department of Respiratory Medicine, Xuzhou Central Hospital of Southeast University, Xuzhou, China.

**Keywords:** apatinib, bevacizumab, cavitary, cystic, lung cancer, thin-walled

## Abstract

**Rationale:**

Asymptomatic, isolated, and thin-walled cystic lung cancer with extensive extrapulmonary metastasis is rare, and the risk of pulmonary cyst developing into lung cancer is poorly understood. The efficacy of apatinib for end-stage pulmonary adenosquamous carcinoma has not been clarified yet.

**Patient concerns:**

We herein report a rare case of primary lung cancer that appeared as an isolated thin-walled cystic lesion on computed tomography (CT) image, who was initially misdiagnosed as having pulmonary cyst empirically.

**Diagnoses:**

Fluorine-18-fluorodeoxyglucose**-**positron emission tomography and CT-guided liver biopsy of the patient revealed extra-pulmonary metastasis of lung cancer.

**Interventions:**

Eight cycles of cisplatin-based chemotherapy were administered, followed by oral apatinib for 6 months. Thereafter, best supportive care was given for this patient.

**Outcomes:**

The pulmonary cystic lesion indicated stable disease through the therapy, but the hepatic tumors were progressed gradually after anticancer treatment. The patient died 16 months after the correct diagnosis.

**Lessons:**

Solitary thin-walled cystic lung cancer should be kept in mind during the differential diagnosis of pulmonary cavitary lesions. Chest CT alone is insufficient for surveillance of these cystic diseases. Timely biopsy and resection are essential to avoid delayed management. Besides, apatinib may play a role in the treatment of end-stage pulmonary adenosquamous carcinoma.

## 1 Introduction

The knowledge about the clinical features of thin-walled cavitary lung cancer is limited for its rarity. It is characterized by cystic malignancy with a wall of ≤4 mm thick, along with at least 75% of the circumference of the lesion.^[1]^ Early lung cancers with cystic airspaces are increasingly being recognized as a cause of delayed diagnoses^[2]^ because solitary thin-walled cavitary lung cancers mimic benign emphysematous diseases, which could be a pitfall in differential diagnosis.^[3]^ In general, the prognosis of patients with cavitary adenocarcinoma is unfavorable.^[4]^

The initial presentation of solitary thin-walled cavity lung cancer varies. It is sometimes difficult to obtain pathological diagnosis from thin-walled cavitary lesions by percutaneous biopsy. The differential diagnosis of benign diseases and malignancies is essential for treatment,^[5]^ besides, solitary thin-walled cavity lung cancer is easily to be neglected by pitfalls in computed tomography (CT) diagnosis. The patients of this kind would take the risk of delayed treatment because it might disseminate without noticeable manifestations. Therefore, a minimally invasive surgery could be considered for patients with high-risk factors for lung cancer.

Treatment options for patients with advanced lung cancer that is resistant to conventional chemotherapy are limited. Apatinib, a small-molecule inhibitor of vascular endothelial growth factor receptor-2, is an orally bioavailable agent for a variety of solid tumors. It has shown a survival benefit in non-small cell lung cancer (NSCLC) in a phase II trial.^[6]^ The efficacy of apatinib for end-stage pulmonary adenosquamous carcinoma has not been clarified yet.

Herein, a rare case of solitary cystic lung cancer with extensive extrapulmonary metastasis is presented, who has missed a timely operation because of the initial misdiagnosis. Related literature is reviewed, with the aim to promote the identification of solitary thin-walled cavitary lung cancer.

## 2 Case presentation

**Figure 1:**
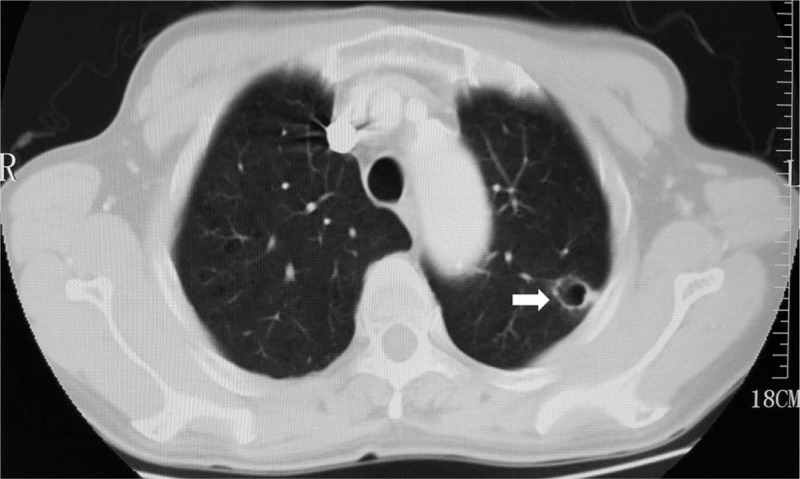
Lung-window computed tomographic image of the patient indicated an isolated thin-walled cystic lesion in left upper lobe (arrow) on November 11^th^, 2015.

A 63-year-old man was admitted to our hospital on June 6^th^, 2016, because of lower back pain for a month, without fever, cough, hemoptysis, hoarseness, or obvious loss of weight. He had no alcohol or tobacco history before admission. His family and social histories were unremarkable. The patient was initially diagnosed as asymptomatic pulmonary cyst empirically on November 11^th^, 2015 during health examination, as his chest CT indicated an isolated thin-walled cystic lesion measuring 1.5 cm in diameter in the left upper lung (Fig. 1). Whole-body CT scan, biopsy, or thoracoscopic resection of the lesion was not performed, and he was advised to take periodic examination by the clinicians in the local hospital.

**Figure 2:**
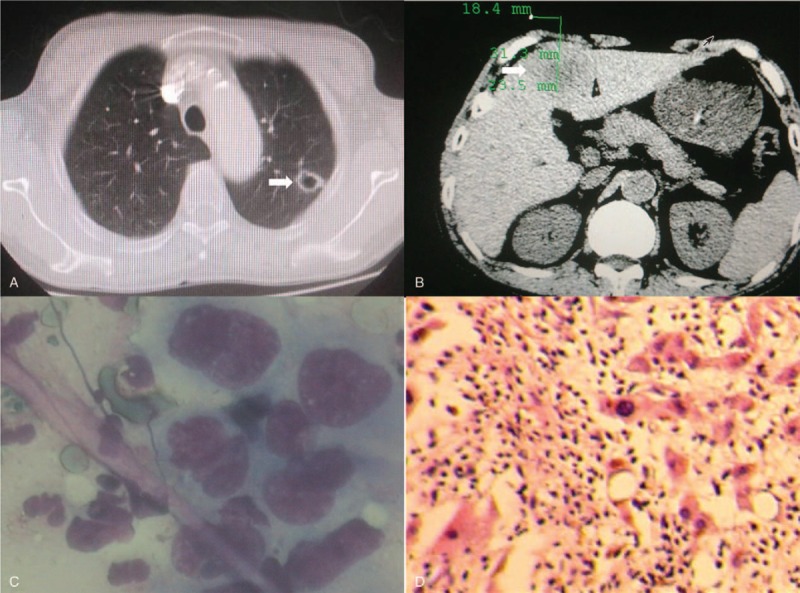
Computed tomography revealed an isolated pulmonary thin-walled cystic lesion (A) and irregular liver masses (B) on June 6^th^, 2016. Liver biopsy displayed atypical malignant cells (C), and it was pathologically confirmed as pulmonary sarcomatoid carcinoma (D), by hematoxylin and eosin staining (×200).

His thorough physical examination on admission showed nothing abnormal. Further tests were performed step by step for differential diagnosis. Routine serum tumor markers of carcinoembryonic antigen, cytokeratin 19 fragment, squamous cell carcinoma, neuron-specific enolase, alpha fetal protein, serum ferritin, carbohydrate antigens (CA) such as CA242, CA72–4, CA153, CA125. and CA19–9 were all in normal range. Subsequently, radiological examinations were carried out for a definite diagnosis. His chest and abdomen CT revealed a morphologically solitary, thin-walled cavitary lesion, measuring 1.6 cm in diameter, along with several hepatic masses (Fig. 2). The cystic lesion was suspicious of malignancy,because the wall was slightly thickened unevenly comparing with the imaging findings (Fig. 1) nearly half a year ago.

**Figure 3:**
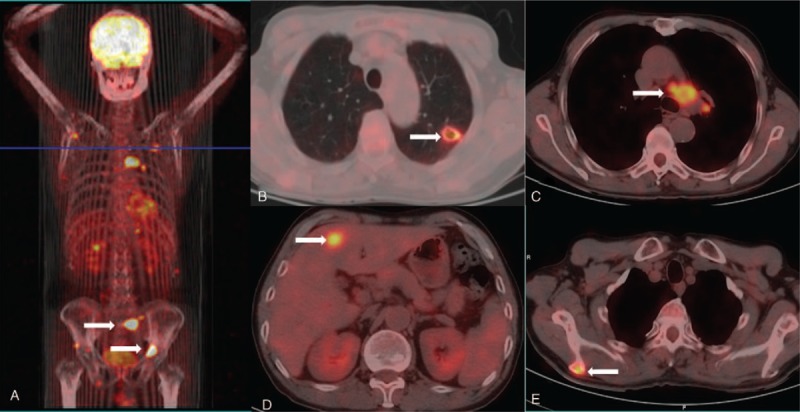
Positron emission tomography-computed tomography of the patient showed a small pulmonary cystic lesion, multiple hepatic masses, intramuscular and osteolytic damages (arrow) on June 6^th^, 2016, with abnormal uptake of Fluorine-18-fluorodeoxyglucose.

Therefore, positron emission tomography-computed tomography (PET-CT) was performed, which indicated a solitary thin-walled pulmonary cystic lesion, several hepatic masses, intramuscular and osteolytic damages, and enlarged mediastinal lymph nodes with hyper-metabolic features. These lesions demonstrated significantly abnormal uptake of fluorine-18-fluorodeoxyglucose (FDG) (Fig. 3). The isolated thin-walled cavitary lesion showed a maximum standard uptake values (SUVmax) of 4.3. Similarly, SUVmax of the masses located in left hepatic lobe, the right scapula, pelvis, and sacrum was 5.6 and 11.3, respectively. The SUVmax of right paratracheal, aortopulmonary, and hilar lymph nodes was 8.4. These lesions were strongly suspicious of malignancy. Then CT-guided percutaneous liver biopsy was performed, which showed aggregation of atypical malignant cells, in accordance with lung cancer (Fig. 2C and D). His Eastern Cooperative Oncology Group (ECOG) score was 1. Based on the above findings, his diagnosis was corrected as stage IV pulmonary adenosquamous carcinoma according to the 7^th^ edition of the TNM staging system for lung cancer.

Subsequently, the patient received 4 cycles of pemetrexed (500 mg/m^2^ of body surface area) plus cisplatin (75 mg/m^2^ of body surface area), followed by 4 cycles of concurrent gemcitabine (1000 mg/m^2^, day 1 and day 8) plus cisplatin (75 mg/m^2^) and bevacizumab (Avastin, Roche Pharma [Schweiz] Ltd., 10 mg/kg of body weight). Meanwhile, zoledronic acid (4 mg at a time) was administered every 21 days along with the chemotherapy. During the treatment, whole-body CT and bone emission computed tomography scan were carried out every 2 to 3 months. The pulmonary cystic lesion indicated stable disease, whereas the hepatic lesions were slightly enlarged after the chemotherapy, as shown by whole-body CT. One month later, he had been recovered from chemotherapy-related thrombocytopenia and fatigue. Then his ECOG score was 2. Oral apatinib (425 mg per day) was given as third-line therapy for 3 months, followed by leukopenia, thrombocytopenia, and cough, which could be controlled by medication. Thereafter, the dosage of apatinib was decreased to 200 mg/day for another 3 months.

**Figure 4:**
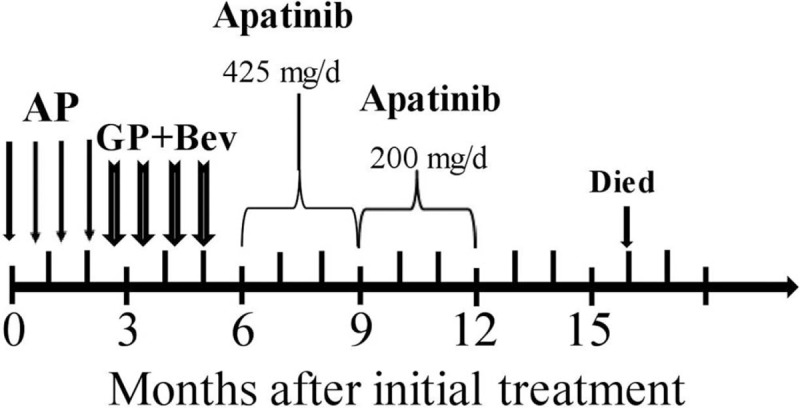
The schematic illustration of therapeutic regimen of the patient. AP = pemetrexed + cisplatin, Bev = bevacizumab, GP = gemcitabine + cisplatin.

The pulmonary cystic lesion maintained stable disease, whereas the hepatic lesions were enlarged and disseminated (progressive disease) as indicated by radiography 15 months after the treatment. Further therapeutic regime was suspended because of concomitant apatinib-related side effects, including discontinuous rhinorrhagia, leukopenia, thrombocytopenia, albuminuria, and fatigue. And his ECOG score was 3 at that time. Therefore, best supportive care was given with the aim to alleviate his suffering, and further laboratory or imaging examinations were no longer performed. His treatment process was depicted in Figure 4. He died of multiple organ failure nearly a month later.

## 3 Discussion

In the era of precision medicine, a timely and accurate diagnosis is the most important premise. Patients with both pulmonary bulla and lung cancer have poor prognosis because they always receive treatment when the tumor is at an advanced stage. Moreover, cavitary tumors tend to be associated with a worse prognosis as compared with noncavitary adenocarcinoma.^[4]^ The features of cystic airspaces include emphysematous bullae, congenital or fibrotic cysts, subpleural blebs, bronchiectatic airways, and distended distal airspaces.^[2]^ However, the prevalence and risk of pulmonary cyst developing into lung cancer are still poorly understood. The patient in our report was misdiagnosed as solitary pulomonary cyst because of lacking knowledge of solitary thin-walled cystic lung cancer. Unfortunately, he missed a timely surgery half a year before his admission, which might deliver a better prognosis for him before the extensive metastasis. Herein, there are several issues about thin-walled cystic lung cancer that are urgently needed to be elucidated.

First, thorough workup for etiologies of cavitary lung lesions is needed to decrease missed and delayed diagnosis. Individuals with chronic obstructive pulmonary disease and emphysema might have a higher frequency of lung cancer.^[7,8]^ Evidence suggests that emphysema is an independent risk factor for lung cancer, and it may be a contributing factor to the development of malignancy.^[9]^ The bronchial wall damage leads to the formation of valves and cavity,^[10]^ and cystic airspaces preceded by nodules can evolve into lung cancer.^[11]^ An isolated cystic airspace with progressive wall thickening over time should raise the suspicion of lung cancer.^[12]^ Location in the periphery of the upper lobes, emphysema, additional cystic lesions or ground-glass nodules, lymphadenopathy, and previous lung cancer should further increase suspicion.^[11]^ A report of 26 cases shows that, in the cystic lesions with initially uniformly thin wall of approximately 1 mm, nodules have been emerged 12 to 118 months (median, 35 months) after the initial CT scan.^[12]^ Another report shows that the median time between the first observation of a cystic airspace and lung cancer diagnosis is 25.5 months.^[11]^ Thus, cystic airspaces with wall thickening and/or associated nodules of any attenuation warrant regular surveillance.

Second, cystic airspaces may be formed after obstruction of the small airways, lepidic growth of adenocarcinoma in emphysema, cystification of tumor because of degeneration, or adenocarcinoma growing along the wall of a preexisting bulla.^[11]^ Cavitary lung nodules could be caused by primary pulmonary cancer, metastatic pulmonary cancer, pulmonary abscess, tuberculosis, fungal infection, infected bulla, coccidioidomycosis, and septic emboli, but the differential diagnosis is sometimes difficult.^[13]^ Furthermore, the notch and irregular internal wall are more frequent in malignant cavitary nodules, whereas a linear margin, presence of satellite nodule, bronchial wall thickening, consolidation, and ground-glass attenuation are more frequent in benign nodules.^[13]^

Specifically, thick-walled cavities (cavity wall thickness >4 mm) are formed as a result of vascular necrosis and destruction of the pulmonary alveoli by excessive mucus through a check-valve mechanism,^[14–16]^ whereas thin-walled cavitary (cavity wall thickness ≤4 mm) lung metastasis can occasionally result from drainage of necrotizing tumor cells via a peripheral bronchus.^[17]^ The thick-walled lung adenocarcinoma patients have a higher frequency of solid predominant tumors, hematogenous metastasis, large-vessel and lymphatic invasion, and necrosis, whereas the lepidic and papillary predominant patterns are more common in the thin-walled patients.^[18]^ Meanwhile, patients with thick-walled cavitary adenocarcinomas have significantly higher incidence of postoperative recurrence and shorter survival, as compared with thin-walled cases, with similar frequency of EGFR and KRAS mutations in both groups.^[18]^ However, the patient in this report with single thin-walled cycstic lung cancer demonstrates extensive hematogenous metastasis, large-vessel and lymphatic invasion, which is inconsistent with the previous reports.

Previously reported cases of thin-walled cystic lung cancer include adenocarcinomas, bronchioalveolar carcinoma,^[19,20]^ squamous cell carcinoma, adenosquamous cell carcinoma,^[15]^ and solitary metastasis of extra-pulmonary tumors.^[17]^ Spontaneous formation of cavity in metastatic lung lesions is rare.^[21]^ Percutaneous needle washing and aspiration of cavitary lesions for cytological examination may be useful for diagnosis.^[22]^ Radiological features including single thin-walled cavity accompanied by uneven thickening of the cavity wall or wall nodules, increased SUV by FDG-PET, and compartments in the cavity on CT images probably indicate lung cancer.^[10]^ However, FDG-PET is useful for workup of lesions with a solid component >8 mm.^[11]^ Progressive wall thickening or appearance/increase of a nodule inside or outside a cystic airspace should raise suspicion of lung cancer irrespective of FDG uptake.^[23]^ Pericystic cancers are reported to be morphologically classified into 4 types as shown in CT images: solid nodule protruding externally (type I) or internally (type II) from the cyst wall, circumferential thickening of the cyst wall (type III), and tissue intermixed within clusters of cysts (type IV).^[2]^ Type I and IV cystic lung cancers are more likely to be misdiagnosed as benign lesions, whereas types II and III cases could easily be confused with inflammation.^[2]^ The case in our report could be classified as type I according to this classification system.

Third, malignancy in cystic lesions may be because of ventilation, clearance, and deposition of carcinogens.^[12]^ Tumor metastasis is an inefficient process, and the major sites of non-small cell lung cancer (NSCLC) metastasis are brain, bone, adrenal gland, and the liver.^[24,25]^ In addition to biopsy, circulating tumor cells (CTCs) or tumour DNA (ctDNA) obtained from peripheral blood may be another diagnostic tool for morphologically atypical lung cancer.^[26]^ Besides, human epidermal growth factor receptor 2, V-Ki-ras2 Kirsten rat sarcoma viral oncogene homolog, and epidermal growth factor receptor mutation status might be detected in CTCs.^[27]^ Lung cancer screen for patients with a giant bulla is necessary. However, it is difficult to obtain sufficient material from thin-walled cavitary lesions by fine needle biopsy; therefore, a timely resection of the bulla is reasonable to avoid delayed diagnosis of coexisting cancer and bulla, especially for patients with high-risk factors for lung cancer.

Similar reports of solitary thin-walled cavity lung cancer are collected (Table 1),^[3,5,11,12,18,22,23,28,29]^ which indicates that distant metastasis may occur when the solitary thin-walled cystic lesion remains unchanged or changed slightly. A few of these collected cases demonstrates distal metastasis with single small thin-walled cystic lung cancer. Therefore, chest CT for annual screening is insufficient because distal dissemination of the malignancy could be asymptomatic. Whole-body CT or FDG-PET is necessary, although PET is not covered by the health insurance in China. Thus, the role of thoracoscopic surgery as a minimally invasive approach is truly indispensable.

**Table 1:**
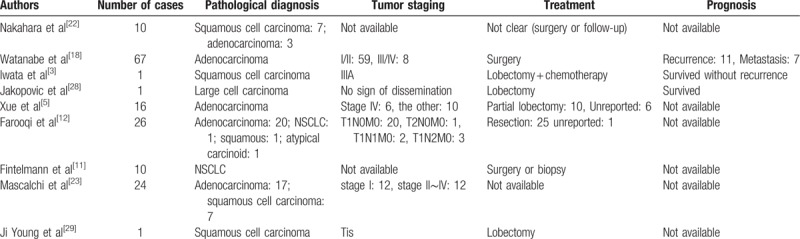
A collection of reports of solitary thin-walled cavity lung cancer.

Finally, the prognosis for patients with progressive or relapsed NSCLC remains poor, regardless of the progress in anticancer agents. First-line therapy is platinum-based regimens, whereas second-line therapies include docetaxel, pemetrexed, and erlotinib. Treatment options for patients who have failed to conventional chemotherapy are limited, and apatinib is considered to be third-line therapy and beyond for NSCLC patients.^[30]^ There has been little progress in targeted therapies for adenosquamous carcinoma, and genetic profiling may facilitate the personalized treatments.^[31]^ Bevacizumab and apatinib inhibit the angiogenesis in malignant tumors, and anti-angiogenesis therapy is a major option for stage IV non-squamous NSCLC patients.^[32]^ As for the patient in this report, apatinib had been administered as third-line therapy for 6 months. Although it demonstrated satisfactory efficacy, many noticeable side effects were also inevitable, which made the patient suffered. The patient in this report survived only 16 months after his corrected diagnosis. More effective target therapeutic agents with decreased adverse reactions are still urgently needed.

## 4 Conclusion

In summary, asymptomatic solitary thin-walled cystic lung cancer should be kept in mind during the differential diagnosis of cavitary lesions, and chest CT alone is inadequate for surveillance of these patients. Thoracoscopic resection could be considered if whole-body CT scan excludes metastasis. A combination of chemotherapy and target therapy is somewhat effective for patients with advanced lung cancer.

## Author contributions

**Data curation:** Xiang Wang, Yun-Xia Tao, Miao Zhang, Wen-Bin Wu.

**Formal analysis:** Yun-Xia Tao.

**Methodology:** Xiang Wang, Yun-Xia Tao, Dun-Peng Yang.

**Resources:** Miao Zhang, Wen-Bin Wu.

**Validation:** Xiang Wang, Min Wang, Dun-Peng Yang.

**Visualization:** Wen-Bin Wu.

**Writing – original draft:** Xiang Wang, Min Wang.

**Writing – review & editing:** Miao Zhang, Min Wang, Wen-Bin Wu, Dun-Peng Yang.
